# Comment on ‘Association Between Handgrip Strength and Cardiovascular Disease Risk in MASLD: A Prospective Study From UK Biobank’ by T. S. Lim et al.—Authors' Reply

**DOI:** 10.1002/jcsm.70075

**Published:** 2025-09-23

**Authors:** Tae Seop Lim, Sujin Kwon, Sung A Bae, Hye Yeon Chon, Seol A. Jang, Ja Kyung Kim, Chul Sik Kim, Seok Won Park, Kyoung Min Kim

**Affiliations:** ^1^ Department of Internal Medicine Yonsei University College of Medicine Seoul Republic of Korea; ^2^ Department of Gastroenterology, Internal Medicine Yongin Severance Hospital, Yonsei University Health System Yongin Republic of Korea; ^3^ Department of Endocrinology, Internal Medicine Yongin Severance Hospital, Yonsei University Health System Yongin Republic of Korea; ^4^ Department of Cardiology, Internal Medicine Yongin Severance Hospital, Yonsei University Health System Yongin Republic of Korea

Dear Editor,

We appreciate the thoughtful comments from Zhao et al. [1] regarding our recent publication [2]. Their insights offer valuable opportunities to clarify aspects of our methodology and further contextualize our findings.

First, with regard to the use of the fibrosis‐4 (FIB‐4) index to define advanced liver fibrosis, we acknowledge its diagnostic limitations, which were also addressed in the limitations section of our manuscript. We applied FIB‐4 not as a diagnostic gold standard, but as a practical, widely used and guideline‐endorsed tool for noninvasive risk stratification in population‐based studies [3, 4, 5, 6, 7]. Although its diagnostic accuracy may vary with factors such as age [8], FIB‐4 remains a validated surrogate for liver fibrosis risk, particularly in settings where liver biopsy or elastography is not feasible [9, 10, 11]. In our study, FIB‐4 was not part of the primary analysis but was used in a subgroup analysis to examine whether the observed association between handgrip strength (HGS) and cardiovascular disease (CVD) risk was maintained irrespective of liver fibrosis severity.

Second, we acknowledge the heterogeneous nature of metabolic dysfunction‐associated steatotic liver disease (MASLD), which spans a spectrum of hepatic and cardiometabolic abnormalities including inflammation, fibrosis and comorbidities such as diabetes mellitus, hypertension and dyslipidaemia [12]. Our primary aim was to evaluate the association between HGS and CVD risk in individuals with MASLD, rather than to characterize histologic or metabolic heterogeneity. To account for key clinical differences, we applied exact propensity score matching across major demographic and metabolic variables. Furthermore, covariates such as body mass index, diabetes mellitus, hypertension, dyslipidaemia and physical activity were also included in our multivariable models. After applying propensity score matching and adjusting covariates, the association between lower HGS and increased CVD risk remained consistent, strengthening the credibility of our findings.

Third, Zhao et al. appropriately emphasize the distinction between association and causation. We fully agree with the comment that causality cannot be determined from observational cohort data alone. In this study, we insisted on the associations between low HGS and CVD risks, not causality between the two conditions, and the findings support the hypothesis that reduced muscle strength may serve as a clinically meaningful and potentially modifiable risk marker. We agree that future studies using methods such as Mendelian randomization would be valuable to clarify causal pathways [13].

Fourth, the concern regarding the long follow‐up period (median 13.1 years) and potential changes in clinical parameters over time is well taken. We acknowledge that repeated measurements of liver function, HGS and lifestyle factors would offer more temporal precision. However, as with many large‐scale cohort studies, our analysis relied on baseline measurements of muscle strength. Although one‐time baseline measurement may not fully capture its temporal variation, the consistency of associations across multiple models supports the notion that baseline HGS may serve as a stable proxy for long‐term risk—forming the basis of our hypothesis. Future studies incorporating repeated assessments of HGS and related clinical variables would help elucidate dynamic relationships with cardiovascular outcomes.

Lastly, we recognize that the generalizability of our findings may be limited by the predominantly European ancestry of the UK Biobank cohort. Nonetheless, our findings are consistent with prior reports from diverse populations and support the growing body of evidence linking sarcopenia to cardiometabolic health [9, 14, 15].

In conclusion, we appreciate the constructive feedback from Zhao et al. and their recognition of the importance of our research. Our study highlights the relevance of muscle strength as an independent cardiovascular risk indicator in MASLD—one that may help improve risk stratification beyond traditional metabolic parameters. We hope our findings contribute to future efforts to develop more personalized preventive strategies in this increasingly prevalent disease population.

## Conflicts of Interest

The authors declare no conflicts of interest.

## References

[1] M. Zhao and J. Zhao , “Comment on ‘Association Between Handgrip Strength and Cardiovascular Disease Risk in MASLD: A Prospective Study From UK Biobank’ by Lim et al,” Journal of Cachexia, Sarcopenia and Muscle 16 (2025): e13854. 40035094 10.1002/jcsm.13757 PMC11876860

[2] T. S. Lim , S. Kwon , S. A. Bae , et al., “Association Between Handgrip Strength and Cardiovascular Disease Risk in MASLD: A Prospective Study From UK Biobank,” Journal of Cachexia, Sarcopenia and Muscle 16 (2025): e13757. 40035094 10.1002/jcsm.13757 PMC11876860

[3] EASL Clinical Practice Guidelines on Non‐Invasive Tests for Evaluation of Liver Disease Severity and Prognosis—2021 Update,” Journal of Hepatology 75 (2021): 659–689. 34166721 10.1016/j.jhep.2021.05.025

[4] M. E. Rinella , B. A. Neuschwander‐Tetri , M. S. Siddiqui , et al., “AASLD Practice Guidance on the Clinical Assessment and Management of Nonalcoholic Fatty Liver Disease,” Hepatology 77 (2023): 1797–1835. 36727674 10.1097/HEP.0000000000000323 PMC10735173

[5] EASL‐EASD‐EASO Clinical Practice Guidelines on the Management of Metabolic Dysfunction‐Associated Steatotic Liver Disease (MASLD),” Journal of Hepatology 81 (2024): 492–542. 38851997 10.1016/j.jhep.2024.04.031

[6] M. N. Kim , J. W. Han , J. An , et al., “KASL Clinical Practice Guidelines for Noninvasive Tests to Assess Liver Fibrosis in Chronic Liver Disease,” Clinical and Molecular Hepatology 30 (2024): S5–s105. 39159947 10.3350/cmh.2024.0506 PMC11493350

[7] F. Kanwal , B. A. Neuschwander‐Tetri , R. Loomba , and M. E. Rinella , “Metabolic Dysfunction‐Associated Steatotic Liver Disease: Update and Impact of New Nomenclature on the American Association for the Study of Liver Diseases Practice Guidance on Nonalcoholic Fatty Liver Disease,” Hepatology 79 (2024): 1212–1219. 38445559 10.1097/HEP.0000000000000670 PMC13435502

[8] S. McPherson , T. Hardy , J. F. Dufour , et al., “Age as a Confounding Factor for the Accurate Non‐Invasive Diagnosis of Advanced NAFLD Fibrosis,” American Journal of Gastroenterology 112 (2017): 740–751. 27725647 10.1038/ajg.2016.453 PMC5418560

[9] E. Han , Y. H. Lee , Y. D. Kim , et al., “Nonalcoholic Fatty Liver Disease and Sarcopenia Are Independently Associated With Cardiovascular Risk,” American Journal of Gastroenterology 115 (2020): 584–595. 32141917 10.14309/ajg.0000000000000572

[10] F. Baratta , D. Pastori , F. Angelico , et al., “Nonalcoholic Fatty Liver Disease and Fibrosis Associated With Increased Risk of Cardiovascular Events in a Prospective Study,” Clinical Gastroenterology and Hepatology 18 (2020): 2324–2331.e4. 31887443 10.1016/j.cgh.2019.12.026

[11] J. Vieira Barbosa , S. Milligan , A. Frick , et al., “Fibrosis‐4 Index Can Independently Predict Major Adverse Cardiovascular Events in Nonalcoholic Fatty Liver Disease,” American Journal of Gastroenterology 117 (2022): 453–461. 35041626 10.14309/ajg.0000000000001606

[12] Z. M. Younossi , M. Kalligeros , and L. Henry , “Epidemiology of Metabolic Dysfunction‐Associated Steatotic Liver Disease,” Clinical and Molecular Hepatology 31 (2025): S32–s50. 39159948 10.3350/cmh.2024.0431 PMC11925440

[13] C. Zhuo , J. Zhao , Q. Wang , et al., “Assessment of Causal Associations Between Handgrip Strength and Cardiovascular Diseases: A Two Sample Mendelian Randomization Study,” Frontiers in Cardiovascular Medicine 9 (2022): 930077. 35990959 10.3389/fcvm.2022.930077 PMC9386423

[14] H. S. Chun , M. N. Kim , J. S. Lee , et al., “Risk Stratification Using Sarcopenia Status Among Subjects With Metabolic Dysfunction‐Associated Fatty Liver Disease,” Journal of Cachexia, Sarcopenia and Muscle 12 (2021): 1168–1178. 34337887 10.1002/jcsm.12754 PMC8517359

[15] K. Y. Choi , T. Y. Kim , Y. E. Chon , et al., “Impact of Anthropometric Parameters on Outcomes in Asians With Metabolic Dysfunction‐Associated Fatty Liver Disease,” Journal of Cachexia, Sarcopenia and Muscle 14 (2023): 2747–2756. 37881112 10.1002/jcsm.13351 PMC10751424

